# Detecting Temporal Cognition in Text: Comparison of Judgements by Self, Expert and Machine

**DOI:** 10.3389/fpsyg.2018.02037

**Published:** 2018-10-26

**Authors:** Erin I. Walsh, Janie Busby Grant

**Affiliations:** ^1^Centre for Research on Ageing, Health & Wellbeing, Australian National University, Canberra, ACT, Australia; ^2^Centre for Applied Psychology, University of Canberra, Canberra, ACT, Australia

**Keywords:** temporal cognition, Stanford Natural Language Parser, self-report, temporal orientation, tense extraction

## Abstract

**Background:** There is a growing research focus on temporal cognition, due to its importance in memory and planning, and links with psychological wellbeing. Researchers are increasingly using diary studies, experience sampling and social media data to study temporal thought. However, it remains unclear whether such reports can be accurately interpreted for temporal orientation. In this study, temporal orientation judgements about text reports of thoughts were compared across human coding, automatic text mining, and participant self-report.

**Methods:** 214 participants responded to randomly timed text message prompts, categorically reporting the temporal direction of their thoughts and describing the content of their thoughts, producing a corpus of 2505 brief (1–358, *M* = 43 characters) descriptions. Two researchers independently, blindly coded temporal orientation of the descriptions. Four approaches to automated coding used tense to establish temporal category for each description. Concordance between temporal orientation assessments by self-report, human coding, and automatic text mining was evaluated.

**Results:** Human coding more closely matched self-reported coding than automated methods. Accuracy for human (79.93% correct) and automated (57.44% correct) coding was diminished when multiple guesses at ambiguous temporal categories (ties) were allowed in coding (reduction to 74.95% correct for human, 49.05% automated).

**Conclusion:** Ambiguous tense poses a challenge for both human and automated coding protocols that attempt to infer temporal orientation from text describing momentary thought. While methods can be applied to minimize bias, this study demonstrates that researchers need to be wary about attributing temporal orientation to text-reported thought processes, and emphasize the importance of eliciting self-reported judgements.

## Introduction

Research into how we cognitively create and experience events from the past and future has become ever more popular in the last decade (e.g., Gardner et al., 2012; Schacter et al., 2012; Stawarczyk and D’Argembeau, 2015; Karapanagiotidis et al., 2017). This work highlights the central role of temporal recall and projection in building and maintaining our self-concept over time, our capacity to appropriately defer short-term gratification for longer-term planning, and manage the complexities of everyday functioning in society (Boyer, 2008; Miloyan et al., 2016; Schacter et al., 2017). While there have been calls for use of more diverse research approaches in the field, to assess thought, behavior and potential interventions in real-world contexts (Oettingen, 2012; Busby Grant and Walsh, 2016; O’Neill et al., 2016), methodological limitations have often restricted when, where and how research into temporal cognition can be conducted.

The majority of studies to date examine temporal thought and associated behavior in controlled lab-based settings. These studies provide insight into the neurological processes underlying past and future thought (e.g., Karapanagiotidis et al., 2017; Thakral et al., 2017), distinctions and relationships between cognitive factors (e.g., Abram et al., 2014; Cole et al., 2016), and the effect of future thought on behavior (e.g., Snider et al., 2016; O’Donnell et al., 2017). While lab-based methodologies provide gold-standard demonstrations of causal effects, they can lack external validity, particularly when they are attempting to demonstrate the efficacy of an intervention on behavior (e.g., Daniel et al., 2015). In contrast, experience sampling allows assessment of thoughts and behaviors in real-world context by prompting participants to report experiences at random intervals during their day. This approach has been demonstrated to provide a scalable, real-world method of assessing temporal thought (Killingsworth and Gilbert, 2010; Song and Wang, 2012; Busby Grant and Walsh, 2016). Diary studies similarly allow participants to report thoughts as experienced in real-world context (Berntsen and Jacobsen, 2008; Finnbogadóttir and Berntsen, 2013), by capturing either spontaneous thoughts, or those responding to cues provided by the researcher (e.g., Gardner et al., 2012). However, these approaches of necessity involve interruption to daily behavior, and can be affected by differential reporting and (in the case of diary studies) retrospective bias.

A different methodology rapidly gaining traction in fields similarly seeking to assess and evaluate human experience is the use of “big data,” in part from social media (Abbasi et al., 2014; Moller et al., 2017; Oscar et al., 2017). This use of existing datasets (e.g., Twitter, Facebook, query logs in Google and Wikipedia, purchasing behavior) rather than active recruitment and data collection has substantial advantages. As well as the sheer size of the data set that can be retrieved, the data has real-world validity because participants are spontaneously recording their own thoughts independent of research context. While there are a number of other challenges around interpretation of this data (e.g., generalisability, differential recording), this approach represents a valuable potential addition to the methodological arsenal which is currently underutilized by psychologists (Oscar et al., 2017).

One of the key challenges for researchers seeking to assess temporal thought using large data sets, such as those created by social media, is the extraction of meaning from relatively small text entries. It is difficult to reliably determine temporal orientation (whether someone is thinking about the past, present or future) from a text statement, particularly in English. Take the statements: “In 2019, I will have remembered this example,” and “I am thinking about making dinner at my parents’ house”; in each of these cases, without the speaker’s own insight to give context, it is not straightforward to identify the temporal orientation. For accurate analysis and interpretation, researchers need to be confident in reliably inferring factors like temporal orientation from a statement, and to take advantage of the large data sets, the analysis needs to take place quickly and accurately, which typically means automated tools rather than manual coding (Cole-Lewis et al., 2015). The focal analysis of Twitter data for human behavior to date has been in sentiment analysis, that is detection of whether a given tweet is positive, negative or neutral relative to a concept, event or product (Oscar et al., 2017; Rosenthal et al., 2017). Numerous machine sentiment classification tools exist, although they differ substantially in their accuracy (Abbasi et al., 2014). To the authors’ knowledge, only Jatowt et al. (2015) and Park et al. (2017) have specifically investigated the temporal orientation of short social media posts (Tweets and facebook statuses, respectively). Jatowt et al. (2015) used the time and date entry identification capacity of the Stanford Natural Language Parser (SNLP) to automatically extract explicit mentions of time (e.g., “tomorrow,” “next month,” “December”). While a highly useful start point that gives insight into the distance in time between the mention (e.g., “last week”) and topic (e.g., “holiday”), this approach is only applicable when explicit mentions of time are present – this is often not the case in natural language, where tense and informational context are the sole cues to orientation. Park et al. (2017) extended this by also including frequency of words in a temporally oriented linguistic enquiry dictionary, but analysis remained constrained to *post hoc* (researcher vs. automated) coding.

The current study is designed to inform researchers seeking to code temporal orientation from existing text data sets, in order to leverage the possibilities of large scale social media corpora for temporal cognition research. This will be achieved by exploring the accuracy of human and automated *post hoc* temporal orientation extraction from real-world short English Language text strings, of the kind found in experience sampling research and on social media microblogging platforms such as Twitter. Careful manipulation of the coding protocol (e.g., allowing single or multiple concurrent possible orientations) and comparison of *post hoc* coding to the participant’s own self-report, rather than potentially innaccurate researcher coding, will provide a useful foundation to set expectations of accuracy in future research.

## Methods

Detailed methods for data collection can be found in Busby Grant and Walsh (2016). Briefly, 214 undergraduate students, aged 17–55 (*M* = 21, *SD* = 7) participated in return for course credit. The sample was 70% female. All participants provided written, informed consent. The ethical aspects of this study were approved by University of Canberra’s Human Research Ethics Committee (protocol 12–134). Participants received 20 text message prompts across 2 days, randomly timed for between 8 am and 8 pm (with some variation of this window on participant request). The high quality random schedules for each participant were generated a-priori using the program “Psrta”. The text messages prompted participants to report the temporal category of their thoughts at the moment the prompt arrived (“What were you thinking about in the seconds before you received the SMS alert?” with options of past/future/present/other), and provide open-ended information about the content of their thoughts (“Please give more information about what you were thinking about in the seconds before you received the SMS alert”).

Participants responded to an average of 14 of the 20 prompts (min = 1, max = 20, *SD* = 6). From an initial corpus of 2884 responses, 379 had either tied (multiple self-selected orientations, despite instructions to produce a statement including only one) or missing self-reported orientation, so were excluded. This resulted in a final corpus of 2505 brief (between 1 and 358 characters, *M* = 43) unique descriptions of momentary temporal thought, from 192 individuals aged 17–52 (*M* = 21.85, *SD =* 6.52), 70% female.

The temporal orientation of unique descriptions of momentary temporal thought was extracted in seven ways, the first being self-report (Table 1). This was followed by *post hoc* human coding by two independent researchers, and automated methods of increasing complexity using the Stanford Natural Language Parser; (SNLP). SNLP coding was undertaken in R version 3.2.0 using the coreNLP package (v 3.3.3) (Manning et al., 2014). Further detail regarding SNLP implementation can be found in Table 1, with full R code available in the Supplementary Materials. Both researcher and SNLP coding was blind to the self-report orientation. For self-report, only one temporal orientation was allowed per description. However, ambiguity in *post hoc* coding can arise from multiple candidate orientations for a single statement. Hence, we also allowed “ties,” circumstances where the either a human or automated coder could specify multiple orientations in an attempt to capture the correct one. These circumstances were coded as “mixed.” Researcher and/or automated coding was considered “correct” when their orientation matched self-report. This is reported as a percentage across the full corpus of 2505 responses. With four possible orientations chance performance was 25%.

**Table 1:** Temporal extraction methods, in the context of the example phrase “In 2019, I will *have remembered this example.*”

Method	Ties	Description	Coding of example phrase	Why this orientation?
(1) Self-rated	No.	This formed a basis for evaluating the remaining methods.	Future	
(2) Researcher A	No	*Post hoc* human coding based on sentence construction and intuition. Here, the researcher must go with their “best guess” when there are multiple candidate orientations.	Future	Sentence context as a whole has cues of future, “will have” and referring to 2019, in the future at time of writing.
(3) Researcher B	Yes	Similar to researcher A, however, in cases where there are multiple candidate orientations, this researcher can select multiple orientations.	Future	Though it is a cue for past orientation, a human reader can see ‘remember’ is used in a future context here.
(4) SNL, naïve	Yes	Automated tense extraction via the Stanford Natural Language Parser using only Penn Treebank POS-tagged word stem cues, with ties allowed. “Future” was marked by modal tense (MD); ‘present’ marked by nouns (NN) present tense verbs (VBG, VBP, VBZ), or interjections (UH); “past” by past tense and participle verbs (VBD, VBN); and “other” by lack of these markers	Mixture, future and past	*In/IN 2019/CD,/, I/PRP will/MD have/VB remembered/VBN this/DT example/NN* both modal tense and past participles present.
(5) SNL, anchor terms	Yes	Uses a combination of the cues used in the naïve method, with additional anchor terms (explicit references to “remembering” and “future”).	Mixture, future and past	with explicit tag of “remember” indicating past tense.
(6) SNL, no ties	No	Builds on the SNL anchor term method but breaks ties by referring to the earliest cue in the sentence.	Future	Future (modal tense MD occurs first).
(7) suTIME	No	As described in Chang and Manning (2012) and applied in similar text mining circumstances by Thorstad and Wolff (2018) and Jatowt et al. (2015), The suTIME tagger of the Stanford Natural Language Parser can be used to extract tense by extracting explicit temporal language (e.g., “Tomorrow,” “Yesterday,” “Today”), or comparing dates from text against when text was created.	Future	*<TIMEX3 tid = “t1” type = “DATE” value = “2019”> 2019</TIMEX3>.*


## Results

**FIGURE 1:**
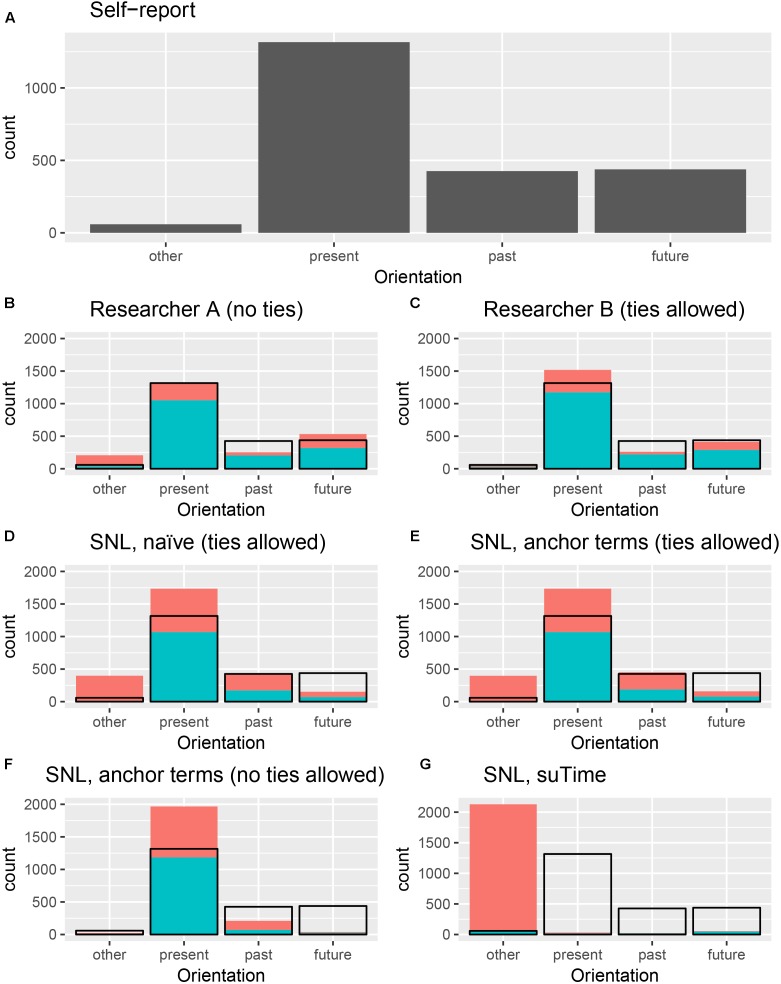
Comparative performance of temporal orientation coding methods. Panel **(A)** shows the distribution of self-reported temporal orientation. For panels **(B)** through **(G)**, black outline denotes distribution of self-reported responses. Blue indicates correspondence between self-report and post hoc coding, red indicates divergence. Counts are not allowed in panels **A** and **F**. Where ties are allowed (panels **C**–**E**,**G**) counts may exceed 2505 (as multiple orientations are possible).

Results are summarized in Figure 1. Text messages were coded based on their temporal directions into the categories as described above: past, present, future, and other. Self-reported orientations indicated the majority (58.78%) of thoughts were oriented to the present. Approximately equal numbers were future- or past- oriented (19.56 and 19.03% respectively), with very few (2.64%) self-categorized as “other” (self-reports in the “other” category were general status reports, such as “sleeping” and “drunk”).

All methods except for suTime (method 7, see Table 1) performed above chance ( > 25% correct). Overall, researcher coding more closely matched self-reported coding than automated methods. When multiple temporal categories per response (ties) were allowed, both researcher and automated methods diverged notably from self-report. Where ties were not allowed, Researcher A (method 2) performed best, with 79.93% correct. Next best was the SNL using both POS-tagged word stems and explicit anchors (method 5), with 57.44% correct. This method notably over-estimated present orientation, particularly at the expense of future orientation. Where ties were allowed, Researcher B (method 3) also outperformed automated methods, with 74.93% correct, and < 1% coded as ties. There was a slight improvement from the naïve to anchored SNL model (48.77 to 49.03%), though both models notably over-estimated both “other” and “present” orientations, at the expense of “future.”

## Discussion

This study highlights the importance of self-report judgements in evaluating accuracy of temporal orientation classification coding systems. The findings demonstrate that, using self-reported orientation as a gold standard, researchers were more accurate than automated systems based on natural language parsers in determining temporal orientation of short text strings. However, the best-performing researcher coding still resulted in around a 20% error rate in temporal orientation classification.

Almost every method (in particular automated methods) overestimated present orientation, and underestimated future orientation. This may be because, in English, present tense can be used to indicate non-present events, and future tense shares similar sentence constructions (Langacker, 2001). For example, “I am thinking about having dinner” could refer to a thought or process coincident with the time of writing (the act of eating dinner) or a future event (a dinner yet to be had). Notably, a recent study similarly extracting temporal orientation from social media text also found a very high degree of present orientation (65% of statements present-oriented in Park et al., 2017). Together with current results, this indicates that present-focus is genuinely the most common temporal thought orientation, so the overestimation seen here may simply be proportional to the number of present vs. future thoughts.

Unexpectedly, attempts to account for bias due to multiple conflicting temporal orientation cues by allowing ties in both human and automated coding led to poorer performance. Too few tied responses were recorded ( < 1%) to determine why human coding performance declined in this method. Broadly, it is likely this relates to a similar phenomenon found in the visual psychophysics and cognitive discrimination literature, which has long recognized that a forced-choice paradigm is peculiarly stable and accurate, possibly by reducing anchoring effects that scale to the number of potential alternative choices (Blackwell, 1952). For automated coding, “ties” were broken by temporal precedence (first cue in the text response was taken as the correct cue). The discrepancy here is therefore most likely due to the “true” temporal cue appearing later in the sentence. Further expansion of the current approach to use the SNL’s parts-of-speech functionality, as in Park et al. (2017), may ameliorate this.

There are a number of implications for researchers seeking to use large data sets to infer and interpret temporal cognition *in situ*. In these cases, self-report of key features such as temporal orientation is generally not available, and researcher coding, while being the most accurate available, is costly and time-consuming and by no means error free. Automated coding of temporal orientation would clearly be the most efficient means of categorizing large text data sets, but the current research highlights the need for further work on appropriate algorithms, using self-report (rather than error-prone researcher coding) as comparison.

This study provides insights into accuracy of temporal coding of text by using a triad of self-report, machine and researcher assessments. It used a substantial corpus of data that closely mirrors the type of data available in big data sets such as social media. However, the sample had limited generalisability (primarily female, undergraduate students) and there is considerable scope for extension to apply substantially more complex algorithms than the SNL tools applied here. There is the possibility of using both automated and researcher coding in concert, given strong historical evidence that a combination of human and automated information processing (human-in-the-loop augmented intelligence) can outperform either alone (Zheng et al., 2017). Further, this paradigm allows a single orientation per description, which may not reflect real-life complexity where multiple orientations are encapsulated within a single chain of thought.

Because the focus of this paper was triangulation of self-report against *post hoc* coding methods, one of the limitations is comparatively unsophisticated automated coding methods. Future research could reduce the gap between human and automated methods through approaches such as machine learning, or tweaking rules to better reflect English structure (e.g., using grammatical, rather than temporal precedence, to break ties, as was done in Park et al. (2017). Such endeavors are underway and ongoing, particularly in the sphere of orientation extraction from social media text (e.g., Park et al., 2017). However, as our results have indicated, reducing the gap between human and automated *post hoc* coding is an important but limited endeavor, as there is also a gap between contemporaneous self-report and *post hoc* researcher coding.

This study explored the accuracy of human and automated *post hoc* temporal orientation extraction, in the context of real-world experiences that sampled English language data. Despite recent advances in natural language parsing, researchers need to be wary about any *post hoc* attribution of temporal orientation to text-reported thought processes, whether human or automated. Our findings demonstrate that future evaluation of the efficacy of automated and machine learning algorithms should use participant’s own, rather than researcher judgement, and emphazise the importance of eliciting self-reported judgements of temporal thought wherever possible.

## Ethics Statement

This study was carried out in accordance with the recommendations of the Australian National University Human Research Ethics Committee with written informed consent from all subjects. All subjects gave written informed consent in accordance with the Declaration of Helsinki. The protocol was approved by the the Australian National University Human Research Ethics Committee (protocol 2012/402).

## Author Contributions

EW contributed to the design of the study, conducted all the statistical analyses, and managed all aspects of the manuscript preparation and submission. JG contributed to the design of the study, provided methodological input and theoretical expertise, advised on statistical analyses, and contributed to writing and editing of the manuscript.

## Conflict of Interest Statement

The authors declare that the research was conducted in the absence of any commercial or financial relationships that could be construed as a potential conflict of interest.
