# Supplementary material for: Detecting Temporal Cognition in Text: Comparison of Judgements by Self, Expert and Machine
Source: Front Psychol. 2018 Oct 26;9:2037. doi: 10.3389/fpsyg.2018.02037 (PMC6212561; doi:10.3389/fpsyg.2018.02037)
Supplement: Supplementary file 1 [file Data_Sheet_1.docx]

#######################################################

# Detecting temporal cognition in text: Comparison of judgements by self, expert and machine

# E.Walsh and J. Busby Grant

# Code: erin.walsh@anu.edu.au

# R version 3.2.0 (2015-04-16)

# Platform: x86_64-w64-mingw32/x64 (64-bit)

# Running under: Windows 7 x64 (build 7601) Service Pack 1

#######################################################

######################## Libraries and functions

# Work

setwd("REMOVED FOR PRIVACY")

# Home

#setwd("REMOVED FOR PRIVACY")

library(psych) # version 1.5.8 for easy descriptives

# coreNLP::downloadCoreNLP() # One-time setup

library(coreNLP) # version 3.3.3Only works at home due to java dependency problems.

library(ggplot2)

library(gridExtra)

# Version 1: naive tense extraction.

snl_tense_extractor_v1<-function(input_text){future<-FALSE;present<-FALSE;past<-FALSE;other<-FALSE

if(grepl("/MD",input_text)){future<-TRUE}

if(grepl("ing/NN",input_text) |

grepl("/VBG",input_text) |

grepl("/VBP",input_text) |

grepl("/VBZ",input_text) |

grepl("/UH",input_text)

){present<-TRUE}

if(grepl("/VBD",input_text) | grepl("/VBN",input_text)){past<-TRUE}

if(!future & !present &!past & !input_text==""){other<-TRUE}

out<-data.frame(text=input_text,future, present,past,other)

return(out)}

# Version 2: naive tense extraction + explicit use of the term 'remember' or 'future' or any stems

snl_tense_extractor_v2<-function(input_text){future<-FALSE;present<-FALSE;past<-FALSE;other<-FALSE

if(grepl("/MD",input_text)){future<-TRUE}

if(grepl("ing/NN",input_text) |

grepl("/VBG",input_text) |

grepl("/VBP",input_text) |

grepl("/VBZ",input_text) |

grepl("/UH",input_text)

){present<-TRUE}

if(grepl("/VBD",input_text) | grepl("/VBN",input_text)){past<-TRUE}

# Addition: any actual reference to 'remembering'

if(grepl("remember",input_text)){past<-TRUE}

# Addition: any actual reference to the future

if(grepl("future",input_text)){future<-TRUE}

if(!future & !present &!past & !input_text==""){other<-TRUE}

out<-data.frame(text=input_text,future, present,past,other)

return(out)}

# Version 3: naive + ties split based on position in sentence, first word wins - allows an 'other' category

snl_tense_extractor_v3<-function(input_text){

input_text<-as.character(input_text)

if(is.na(input_text)){return(NA)}

if(nchar(input_text)==1){return(NA)}

splittit<-paste0("/",unlist(strsplit(input_text,"/")))

futurepos<-c(grep("/MD",splittit),grep("future",splittit))

presentpos<-c(grep("ing/NN",splittit), grep("/NN",splittit),

grep("/VBG",splittit),

grep("/VBP",splittit),

grep("/VBZ",splittit),

grep("/UH",splittit))

pastpos<-c(grep("/VBD",splittit),

grep("/VBN",splittit),

grep("remember",splittit))

# Place back in order so we can pull out the first one

theorder<-data.frame( rbind(

cbind(rep("future", length(futurepos)),futurepos),

cbind(rep("present", length(presentpos)),presentpos),

cbind(rep("past", length(pastpos)),pastpos)))

out<-as.character(theorder[which.min(as.numeric(levels(theorder[,2]))[theorder[,2]]) ,1])

# Make the output dataframe; boolean test or if empty due to missingness coerce to false

future<-out=="future"; if(length(future)==0){future<-FALSE}

past<-out=="past"; if(length(past)==0){past<-FALSE}

present<-out=="present"; if(length(present)==0){present<-FALSE}

other<-(!future & !past & !present)

# Return

outdat<-data.frame(text=input_text, future, present, past, other)

return(outdat)}

#######################################################

######################## Data and checks

# Self-report results

sr_dat<-read.csv("data/self_report.csv")

# Check it loaded correctly

head(sr_dat)

length(unique(as.character(sr_dat$moreinfo))) # 2570 unique entries

# Ensure the 'moreinfo' column is correctly recognised as character, not factor.

sr_dat$moreinfo<-as.character(sr_dat$moreinfo)

# Get rid of empty rows

sr_dat<-sr_dat[-which(sr_dat$moreinfo==""),]

# Check

head(sr_dat); nrow(sr_dat) # 2885

# specify rater

sr_dat$rater<-"Self"

# Remove empty rows

sr_dat<-sr_dat[-which(as.character(sr_dat$moreinfo)==" "),] # 2884

# Collapse redundant categories

sr_dat[which(!is.na(sr_dat$remembering)),"past"]<-TRUE

sr_dat[which(!is.na(sr_dat$knowing)),"past"]<-TRUE

sr_dat[which(!is.na(sr_dat$present)),"present"]<-TRUE

sr_dat[which(!is.na(sr_dat$future)),"future"]<-TRUE

sr_dat[which(!is.na(sr_dat$imagining)),"imagining"]<-TRUE

sr_dat<-sr_dat[,c("moreinfo","past","present","future","other")]

# Researcher coded results

researcher_noties<-read.csv("data/orientation_coding_EW.csv")

# Check it loaded correctly

head(researcher_noties)

length(unique(as.character(researcher_noties$moreinfo))) # 2569 unique entries

# Ensure the 'moreinfo' column is correctly recognised as character, not factor.

researcher_noties$moreinfo<-as.character(researcher_noties$moreinfo)

# Check

head(researcher_noties);nrow(researcher_noties) # 2885

researcher_ties<-read.csv("data/orientation_coding_JBG.csv")

# Check it loaded correctly

head(researcher_ties)

length(unique(as.character(researcher_ties$moreinfo))) # 2569 unique entries

# Ensure the 'moreinfo' column is correctly recognised as character, not factor.

researcher_ties$moreinfo<-as.character(researcher_ties$moreinfo)

# Get rid of empty rows

head(researcher_ties);nrow(researcher_ties) # 2885

# Convert 'NA' to 'FALSE' and 1 to 'TRUE'

researcher_noties[is.na(researcher_noties)] <- FALSE

researcher_noties[,c("Past","Present","Future","Other")]<-researcher_noties[,c("Past","Present","Future","Other")]==1

researcher_ties[is.na(researcher_ties)] <- FALSE

researcher_ties[,c("Past","Present","Future","Other")]<-researcher_ties[,c("Past","Present","Future","Other")]==1

# Allow ties for 'researcher_ties'

researcher_ties$mixed<- rowSums(researcher_ties[,c("Past", "Present", "Future", "Other")])>1

# All rownames to lowercase for later merging

names(researcher_noties)<-tolower(names(researcher_noties))

names(researcher_ties)<-tolower(names(researcher_ties))

# Specify rater

researcher_noties$rater<-"Researcher, no ties allowed"

researcher_ties$rater<-"Researcher,ties allowed"

# Remove empty rows

researcher_noties<-researcher_noties[-which(as.character(researcher_noties$moreinfo)==" "),] # 2884

researcher_ties<-researcher_ties[-which(as.character(researcher_ties$moreinfo)==" "),] # 2884

#######################################################

######################## Parsing 001: Stanford Natural Language Parser

# (URL for manual comparison is http://nlp.stanford.edu:8080/parser/index.jsp)

# Clean to remove untokenizable non-askii characters

to_tokenize<-sr_dat$moreinfo

Encoding(to_tokenize) <- "latin1"

to_tokenize_ascii_cleaned<-iconv(to_tokenize, "latin1", "ASCII", sub="")

# Actually tokenize

# # Last run 27/3/2018

# # Initialize

initCoreNLP()

snl_annotated<-lapply(to_tokenize_ascii_cleaned,annotateString)

snl_tokens<-lapply(snl_annotated,getToken)

snl_tokens_string<-lapply(snl_tokens,function(x){paste0(x$token,"/",x$POS)})

snl_tokens_string_flat<-as.character(unlist(lapply(snl_tokens_string, function(x){paste0(x,collapse=" ")})))

# # Check

head(cbind(sr_dat$moreinfo,snl_tokens_string_flat))

tail(cbind(sr_dat$moreinfo,snl_tokens_string_flat))

# # Save

# save(snl_annotated,snl_tokens,snl_tokens_string,snl_tokens_string_flat, file="data/snl_tokens.RData")

# Load

load("data/snl_tokens.RData")

# Run

snl_tense_extracted_v1<-lapply(snl_tokens_string_flat,snl_tense_extractor_v1)

snl_tense_extracted_v2<-lapply(snl_tokens_string_flat,snl_tense_extractor_v2)

snl_tense_extracted_v3<-lapply(snl_tokens_string_flat,snl_tense_extractor_v3)

# Rbind and clean

snl_v1<-do.call(rbind, snl_tense_extracted_v1)

snl_v1$text<-as.character(snl_v1$text)

snl_v1$rater<-"SNL, naive"

snl_v2<-do.call(rbind, snl_tense_extracted_v2)

snl_v2$text<-as.character(snl_v2$text)

snl_v2$rater<-"SNL, anchor terms"

snl_v3<-do.call(rbind, snl_tense_extracted_v3)

snl_v3$text<-as.character(snl_v3$text)

snl_v3$rater<-"SNL, anchor terms, ties broken by position (first wins)"

######################## SUTime temporal tagger

# As in Thorstad, R., & Wolff, P. (2018). A big data analysis of the relationship between future thinking and decision-making.

# Proceedings of the National Academy of Sciences, 115(8), E1740-E1748.

# Can't run it propely in R, so will use my own code + the web portal at http://nlp.stanford.edu:8080/sutime/process

# Named Entity Recognition, TimeGuidelines for Temporal Expression Annotation for English for TempEval 2010

# Formal citation: Angel X. Chang and Christopher D. Manning. 2012. SUTIME: A Library for Recognizing and Normalizing Time Expressions.

# 8th International Conference on Language Resources and Evaluation (LREC 2012).

snl_ner_string<-lapply(snl_tokens,function(x){paste0(x$token,"/",x$NER)})

snl_ner_string_temps<-grepl("/DATE",snl_ner_string)

just_temporals<-to_tokenize_ascii_cleaned[which(snl_ner_string_temps)]

# Ran this through http://nlp.stanford.edu:8080/sutime/process

just_temporals_tagged<-read.csv("data/su_time.csv")

just_temporals_tagged_unique<-just_temporals_tagged[!duplicated(just_temporals_tagged$token), ]

# Pull out timex3 categories

timex3<-just_temporals_tagged_unique$category

# Strip off explicit dates

timex3_stripped<-gsub('[^a-zA-Z]', '', timex3)

timex3_stripped

# timex3, pull out actual tags

tense<-vector()

for(i in 1:nrow(just_temporals_tagged_unique)){

temp<-strsplit(as.character(just_temporals_tagged_unique$timex3[i]),">")[[1]][2]

tense[i]<-strsplit(as.character(temp),"<")[[1]][1]}

snl_ner_out<-data.frame(moreinfo=sr_dat$moreinfo,

temporal_tags_tense=NA,

temporal_tags_timex3=NA,

temporal_tags_timex3_raw=NA)

just_temporals_tagged_unique$timex3<-as.character(just_temporals_tagged_unique$timex3)

# To keep track if multiple indicators are in same place

indices<-rep(NA,nrow(snl_ner_out))

# Stitch our indicators back on

for(i in 1:length(tense)){

snl_ner_out[which(grepl(tense[[i]],snl_ner_out$moreinfo)),"temporal_tags_tense"]<-tense[[i]]

snl_ner_out[which(grepl(tense[[i]],snl_ner_out$moreinfo)),"temporal_tags_timex3"]<-timex3_stripped[[i]]

snl_ner_out[which(grepl(tense[[i]],snl_ner_out$moreinfo)),"temporal_tags_timex3_raw"]<-just_temporals_tagged_unique$timex3[i]

indices[which(grepl(tense[[i]],snl_ner_out$moreinfo))]<-indices[which(grepl(tense[[i]],snl_ner_out$moreinfo))]+1

}

# Tidy

snl_ner_out$multiples<-indices>1

snl_ner_out$rater<-"snl_SUTIME"

# Where SU time tags were created, swap them in here.

snl_ner_out$moreinfo<-as.character(snl_ner_out$moreinfo)

snl_ner_out[which(!snl_ner_out$temporal_tags_timex3_raw==""),"moreinfo"]<-

snl_ner_out[which(!snl_ner_out$temporal_tags_timex3_raw==""),"temporal_tags_timex3_raw"]

# Does not neatly give past/future, but rather relative days. Replace with our goal format.

table(tolower(snl_ner_out$temporal_tags_tense))

snl_ner_out$temporal_tags_cleaned<-NA

snl_ner_out$temporal_tags_cleaned[which(tolower(snl_ner_out$temporal_tags_tense) %in% c("about 5 minutes ago","past"))]<-"past"

snl_ner_out$temporal_tags_cleaned[which(tolower(snl_ner_out$temporal_tags_tense) %in% c("presently","now","present"))]<-"present"

snl_ner_out$temporal_tags_cleaned[which(tolower(snl_ner_out$temporal_tags_tense) %in% c("future","next week","the future","tomorrow"))]<-"future"

snl_ner_out$temporal_tags_cleaned[which(!tolower(snl_ner_out$temporal_tags_tense) %in%

c("about 5 minutes ago","past",

"presently","now","present",

"future","next week","the future","tomorrow"))]<-"other"

table(tolower(snl_ner_out$temporal_tags_cleaned))

#######################################################

#################################### BASIC COMPARISONS (28/03/2018)

compare_single_response<-function(x){

sr_outcome<-names(sr_dat)[which(sr_dat[x,]==1)]

# If there is no outcome, it should be NA.

if(length(sr_outcome)==0){sr_outcome<-NA}

# If there is more than one self-reported orientation, make this missing.

if(length(sr_outcome)==2){sr_outcome<-NA}

sr_to_bind<-cbind(as.character(sr_dat[x,"moreinfo"]),"Self", sr_outcome)

noties_to_bind<-cbind(researcher_noties[x,"moreinfo"], researcher_noties[x,]$rater,

names(researcher_noties)[which( researcher_noties[x,]==1)])

ties_to_bind<-cbind(researcher_ties[x,"moreinfo"], researcher_ties[x,]$rater,

names(researcher_ties)[which(researcher_ties[x,]==1)])

if(length(noties_to_bind)==2){noties_to_bind<-cbind(noties_to_bind,NA)} # To fix some missingness glitches

if(length(ties_to_bind)==2){ties_to_bind<-cbind(ties_to_bind,NA)} # To fix some missingness glitches

snl_v1_to_bind<-cbind(snl_v1[x,"text"], snl_v1[x,]$rater,names(snl_v1)[which(snl_v1[x,]==1)])

snl_v2_to_bind<-cbind(snl_v2[x,"text"], snl_v2[x,]$rater,names(snl_v2)[which(snl_v2[x,]==1)])

snl_v3_to_bind<-cbind(snl_v3[x,"text"], snl_v3[x,]$rater,names(snl_v3)[which(snl_v3[x,]==1)])

snl_sutime_to_bind<-cbind(snl_ner_out[x,"moreinfo"], snl_ner_out[x,]$rater,snl_ner_out[x,]$temporal_tags_cleaned)

out<-rbind(sr_to_bind,noties_to_bind,ties_to_bind,snl_v1_to_bind,snl_v2_to_bind,snl_v3_to_bind,snl_sutime_to_bind)

rownames(out)<-NULL # To avoid annoying error

out<-data.frame(out)

names(out)<-c("Text/coded","Rater","Orientation")

# Re-order for easier reading

out<-out[,c("Orientation","Rater","Text/coded")]

# See which of the methods match the orientation provided by self-report

out$correct<-out[which(out$Rater=="Self"),"Orientation"] == out[,"Orientation"]

return(out)

}

single_accrual<-list() # Set up accumulator

# There were 2884 unique responses.

for(i in 1:2884){single_accrual[[i]]<-compare_single_response(i)}

# Remove those with missing self-report, as this is the benchmark.

SR_is_missing<- is.na(unlist(lapply(single_accrual,"[", 1, 1)))

table(SR_is_missing)

# SR_is_missing

# FALSE TRUE

# 2505 379

single_accrual_selected<-single_accrual[which(!SR_is_missing)]

length(single_accrual_selected)

# Collapse into seperate dataframes for descriptives and further analysis

code_1_self_rated<-do.call(rbind,lapply(single_accrual_selected,function(x){x[which(x$Rater=="Self"),]}))

table(code_1_self_rated$Orientation);head(code_1_self_rated)

# Researcher

code_2_Researcher_no_ties_allowed<-do.call(rbind,lapply(single_accrual_selected,function(x){x[which(x$Rater=="Researcher, no ties allowed"),]}))

table(code_2_Researcher_no_ties_allowed$Orientation);head(code_2_Researcher_no_ties_allowed)

code_3_Researcher_ties_allowed<-do.call(rbind,lapply(single_accrual_selected,function(x){x[which(x$Rater=="Researcher,ties allowed"),]}))

table(code_3_Researcher_ties_allowed$Orientation);head(code_3_Researcher_ties_allowed)

# SNL

code_4_SNL_naive<-do.call(rbind,lapply(single_accrual_selected,function(x){x[which(x$Rater=="SNL, naive"),]}))

table(code_4_SNL_naive$Orientation); head(code_4_SNL_naive)

snl_tense_extractor_v1("In/IN 2019/CD ,/, I/PRP will/MD have/VB rememberd/VBN this/DT example/NN.")

code_5_SNL_anchor_terms_ties<-do.call(rbind,lapply(single_accrual_selected,function(x){x[which(x$Rater=="SNL, anchor terms"),]}))

table(code_5_SNL_anchor_terms_ties$Orientation); head(code_5_SNL_anchor_terms_ties)

snl_tense_extractor_v2("In/IN 2019/CD ,/, I/PRP will/MD have/VB rememberd/VBN this/DT example/NN.")

code_6_SNL_anchor_no_ties<-do.call(rbind,lapply(single_accrual_selected,function(x){x[which(x$Rater=="SNL, anchor terms, ties broken by position (first wins)"),]}))

table(code_6_SNL_anchor_no_ties$Orientation); head(code_6_SNL_anchor_no_ties)

snl_tense_extractor_v3("In/IN 2019/CD ,/, I/PRP will/MD have/VB rememberd/VBN this/DT example/NN.")

code_7_SNL_sutime<-do.call(rbind,lapply(single_accrual_selected,function(x){x[which(x$Rater=="snl_SUTIME"),]}))

table(code_7_SNL_sutime$Orientation); head(code_7_SNL_sutime)

####################################################### Plots!

grid.arrange(ncol=1, heights=c(0.3,0.7),

ggplot(na.omit(code_1_self_rated), aes(x=Orientation)) + geom_bar() +

theme(legend.position = "none") + ggtitle("1) Self-report")

,

arrangeGrob(

ggplot(na.omit(code_2_Researcher_no_ties_allowed), aes(x=Orientation, fill=correct)) + geom_bar() +

theme(legend.position = "none") + ggtitle("2) Researcher A (no ties)") +

geom_bar(data=code_1_self_rated, aes(x=Orientation),color="black", fill=NA) +ylim(0,2000)

,

ggplot(na.omit(code_3_Researcher_ties_allowed[which(!code_3_Researcher_ties_allowed$Orientation=="mixed"),]), #Omit this for tidy

aes(x=Orientation, fill=correct)) + geom_bar() +

theme(legend.position = "none") + ggtitle("3) Researcher B (ties allowed)") +

geom_bar(data=code_1_self_rated, aes(x=Orientation),color="black", fill=NA)+ylim(0,2000)

,

ggplot(na.omit(code_4_SNL_naive), aes(x=Orientation, fill=correct)) + geom_bar() +

theme(legend.position = "none") + ggtitle("4) SNL, naïve (ties allowed)") +

geom_bar(data=code_1_self_rated, aes(x=Orientation),color="black", fill=NA)+ylim(0,2000)

,

ggplot(na.omit(code_5_SNL_anchor_terms_ties), aes(x=Orientation, fill=correct)) + geom_bar() +

theme(legend.position = "none") + ggtitle("5) SNL, anchor terms (ties allowed)") +

geom_bar(data=code_1_self_rated, aes(x=Orientation),color="black", fill=NA)+ylim(0,2000)

,

ggplot(na.omit(code_6_SNL_anchor_no_ties), aes(x=Orientation, fill=correct)) + geom_bar() +

theme(legend.position = "none") + ggtitle("6) SNL, anchor terms (no ties allowed)") +

geom_bar(data=code_1_self_rated, aes(x=Orientation),color="black", fill=NA)+ylim(0,2000)

,

ggplot(na.omit(code_7_SNL_sutime), aes(x=Orientation, fill=correct)) + geom_bar() +

theme(legend.position = "none") + ggtitle("7) SNL, suTime") +

geom_bar(data=code_1_self_rated, aes(x=Orientation),color="black", fill=NA)

))

####################################################### Percentages!

round(prop.table(table(code_1_self_rated$Orientation))*100,2)

round(prop.table(table(code_2_Researcher_no_ties_allowed$correct))*100,2)

round(prop.table(table(code_3_Researcher_ties_allowed$correct))*100,2)

round(prop.table(table(code_4_SNL_naive$correct))*100,2)

round(prop.table(table(code_5_SNL_anchor_terms_ties$correct))*100,2)

round(prop.table(table(code_6_SNL_anchor_no_ties$correct))*100,2)

round(prop.table(table(code_7_SNL_sutime$correct))*100,2)

# Overall percentages.

round(prop.table(table(c(code_2_Researcher_no_ties_allowed$correct,

code_3_Researcher_ties_allowed$correct))*100),2)

round(prop.table(table(c(code_4_SNL_naive$correct,

code_5_SNL_anchor_terms_ties$correct,

code_6_SNL_anchor_no_ties$correct,

code_7_SNL_sutime$correct))*100),2)

round(prop.table(table(c(code_2_Researcher_no_ties_allowed$correct,

code_6_SNL_anchor_no_ties$correct))*100),2)

# Ties not allowed

round(prop.table(table(code_2_Researcher_no_ties_allowed$correct))*100,2)

round(prop.table(table(code_6_SNL_anchor_no_ties$correct))*100,2)

# Ties allowed

round(prop.table(table(code_3_Researcher_ties_allowed$correct))*100,2)

round(prop.table(table(code_3_Researcher_ties_allowed$correct,

code_3_Researcher_ties_allowed$Orientation))*100,1)

round(prop.table(table(code_4_SNL_naive$correct))*100,2)

round(prop.table(table(code_5_SNL_anchor_terms_ties$correct))*100,2)
